# Two-Component Rare-Earth Fluoride Materials with Negative Thermal Expansion Based on a Phase Transition-Type Mechanism in 50 *R*F_3_-*R*’F_3_ (*R* = La-Lu) Systems

**DOI:** 10.3390/ijms241814000

**Published:** 2023-09-12

**Authors:** Boris P. Sobolev, Elena A. Sulyanova

**Affiliations:** Shubnikov Institute of Crystallography, Federal Scientific Research Centre “Crystallography and Photonics”, Russian Academy of Sciences, Leninskiy Prospekt 59, 119333 Moscow, Russia; sobolev.b@crys.ras.ru

**Keywords:** negative thermal expansion, polymorphic transformation, rare-earth trifluorides, phase diagrams

## Abstract

The formation of materials with *negative thermal expansion* (**NTE**) based on a *phase transition-type mechanism* (**NTE-II**) in 50 ***T****–x* (temperature–composition) *R*F_3_-*R*’F_3_ (*R* = La-Lu) systems out of 105 possible is predicted. The components of these systems are “*mother*” *R*F_3_ compounds (*R* = Pm, Sm, Eu, and Gd) with *polymorphic transformations* (**PolTrs**), which occur during heating between the main structural types of *R*F_3_: ***β***-(β-YF_3_) → ***t***-(*mineral tysonite* LaF_3_). The PolTr is characterized by a *density anomaly*: the *formula volume* (V_form_) of the low-temperature modification (V***_β-_***) is higher than that of the high-temperature modification (V***_t-_***) by a *giant* value (up to 4.7%). In *R*F_3_-*R’*F_3_ systems, isomorphic substitutions *chemically modify R*F_3_ by forming *R*_1−x_*R’*_x_F_3_ *solid solutions* (***ss***) based on both modifications. A *two-phase composite* (***β-ss*** + ***t-ss***) is a *two-component* NTE-II material with *adjustable* parameters. The prospects of using the material are estimated using the parameter of the *average volume change* (ΔV/V_av_). The V_av_ at a fixed gross composition of a system is determined by the ***β-ss*** and ***t-ss*** decay (synthesis) curves and the temperature ***T***. The regulation of ΔV/V_av_ is achieved by changing ***T*** within a “*window* Δ***T***”. The available Δ***T*** values are determined using phase diagrams. A *chemical classification* (**ChCl**) translates the search for NTE-II materials from 15 *R*F_3_ into an array of 105 *R*F_3_-*R*’F_3_ systems. Phase diagrams are divided into 10 *types of systems* (**TypeS**s), in four of which NTE-II materials are formed. The tables of the systems that comprise these TypeSs are presented. The position of ***T***_trans_ of the PolTr on the ***T*** scale for a short *quasi-system* (**QS**) “from PmF_3_ to TbF_3_” determines the interval of the Δ***T***_trans_ offset achievable in the *R*F_3_-*R’*F_3_ systems: from −148 to 1186 ± 10 °C. NTE-II fluoride materials exceed known NTE-II materials by almost three times in this parameter. Equilibrium in *R*F_3_-*R’*F_3_ systems is established quickly. The number of qualitatively different two-component fluoride materials with the *giant* NTE-II can be increased by more than ten times compared to *R*F_3_ with NTE-II.

## 1. Introduction

Trifluorides of rare-earth elements (**REE**), *R*F_3_, are polymorphic [1,2,3]. In [4], the discovery of an effect called a density anomaly in *lanthanide* (***Ln***) trifluorides, *Ln*F_3_, was reported. The anomaly occurs during the PolTr This is because the density of the high-temperature modification is higher than that of the low-temperature modification. This usually happens in the opposite direction. Such an anomaly has been described [4] for *Ln*F_3_ with *Ln* = Sm, Eu, and Gd. At that time, there was no interest in materials with NTE. The publication of [4] went unnoticed. In subsequent studies, there was no mention that any *R*F_3_ other than ScF_3_ [5,6,7] was an NTE material.

The polymorphism of PmF_3_ was not known because all its isotopes are short-lived, and the synthesis of Pm is possible only in atomic reactors. Recently, a method of structural and chemical modeling in a “CeF_3_-GdF_3_” QS was developed [8]. The composition _61_(_58_Ce_0.5_Gd_0.5_)F_3_ with an *atomic number* (**Z**) of promethium, Z = 61, was proposed and named “*pseudo*-PmF_3_”. It has the structural and thermal (PolTr) properties of _61_PmF_3_. “*Pseudo*-PmF_3_” polymorphism attached PmF_3_ to dimorphic *Ln*F_3_ with *Ln* = Pm, Sm, Eu, and Gd, which are materials with NTE-II [9].

In this study, the concept [10] of the *chemical modification* of components of *R*F_3_-*R’*F_3_ (*R* = La-Lu) systems was used to predict 50 *R*F_3_-*R’*F_3_ systems (out of 105 possible) with *two-phase composites* having the *giant* NTE-II at the PolTr.

According to IUPAC, 17 REE were designated as *R* = Sc, Y, or La, and 14 *Ln*. When analyzing the periodicity in the properties of REE compounds, it is necessary to separate the *d*-element La from the 4*f*-elements *Ln*. If not necessary, the sum (La + *Ln*) is designated *R*.

The classification of materials with NTE presented in [10] cannot be considered complete. The authors of this article have written this statement. He identified materials with NTE-I (with NTE in a wide temperature (***T***) interval) and NTE-II (associated with the PolTr in a narrow “*window* Δ***T***”).

To verify the *necessity* and/or *sufficiency* of the empirical features of NTE-II materials [10], it is necessary to verify their compliance with the fundamental rules for constructing equilibrium phase diagrams of chemical systems. The materials formed in the NTE-II systems must comply with thermodynamic rules.

The origin of the features of NTE-II materials is discussed using an example of the phase diagram of this system. Their manifestation in the phase diagram is in line with the rules for constructing phase diagrams.

Such a comparison showed that the common features of NTE-II materials according to [10] (the “*window* Δ***T***” and *two-phase region*) neither separately nor together can determine NTE-II materials.

The *necessary* and *sufficient* signs of NTE-II materials are *polymorphism* and *density anomaly*. The *anomaly* is that the *formula volume* (**V_form_**, the volume of one formula unit) of the low-temperature (***β***-) modification (V_low_) is higher than that of the high-temperature (***t***-) modification (V_high_).

*Chemical modification by isomorphism* of known *R*F_3_ with NTE-II is possible only in a binary (and more complex) system. For trifluorides of *R*, such systems are *R*F_3_-*R*’F_3_. From 17 *R*F_3_, 136 systems are formed. From 15 fluorides (without fluorides of *d-*elements: ScF_3_ has a structure different from *R*F_3_ and YF_3_), 105 systems are formed. They are considered in this work.

Four dimorphic fluorides form the *structural subgroup* (**SSGr**) ***B***: PmF_3_, SmF_3_, EuF_3_, and GdF_3_. They have the PolTr with the *giant* NTE-II (ΔV/V ~ 4.7% relative to the smaller value) [9].

Such an *anomaly* is rare for inorganic compounds. The structural mechanism of the *giant* NTE-II for the ***β***- → ***t***-PolTr (at heating) is unknown.

*R*F_3_-*R’*F_3_ systems are the most suitable for the creation of fluoride NTE-II materials. *R*F_3_ are ionic compounds with the simplest formula and high chemical stability. Their *melting* (***T***_fus_) and PolTr (***T***_trans_) temperatures provide diffusion processes that quickly achieve equilibrium.

A long *homologous series* of LaF_3_ and 14 *Ln*F_3_ (*Ln* = Ce-Lu) consists of extremely *chemically similar* (**ChProx**) compounds with a minimum difference in the Z of cations in the series (ΔZ = 1).

Over the whole length of the series, lanthanide compression of cations [11] leads to the formation of three types of structures: LaF_3_ (***t***-) [12,13], β-YF_3_ (***β***-) [14], and α-UO_3_ (***α***-) [15]. They are separated by two *morphotropic transformations* (**MorphTrs**).

*High-temperature chemical interactions* of the components in the *R*F_3_-*R’*F_3_ systems are described by the ChCl [16]. ChCl translates the search for NTE-II materials from 15 *R*F_3_ to a complete array of 105 *R*F_3_-*R’*F_3_ systems.

ChCl reflects periodic changes in the structure and properties of the *R*F_3_ *homological series* [16] and the systems formed by them. Unlike the “dimensionless” ChProx, the ChCl is based on the measurable parameter ΔZ = |^N^Z−^M^Z|.

The *chemistry of the high-temperature interactions* of components is visually reflected in the *topological features* (forms) of phase diagrams. The most common form of weak interaction in the *R*F_3_-*R*’F_3_ systems is *isomorphism*. Depending on ΔZ, it varies from perfect to limited.

Stronger forms of interactions are two types of *morphotropic transformations*. Their *topological features* are invariant *phase reactions*: *peritectic* (MorphTrs-1) and *eutectic* (MorphTrs-2). The maximum chemical differences between the components and ΔZ (from 8 to 14) are accompanied by the presence of both MorphTr-1 and MorphTr-2 in one system or a complete rupture of the isomorphic miscibility [1].

A *chemical design* of the *R*F_3_-*R’*F_3_ systems covers a complete array of 105 binary systems formed by REE fluorides (without ScF_3_ and YF_3_). It is based on studies of the *chemistry of high-temperature interactions* between components [1].

The structural and chemical modeling of “*pseudo*-_61_PmF_3_” detected the low-temperature “hidden” PolTr in it, gave the values of the lattice parameters, and detected the *giant* NTE-II [9].

The *short* “from PmF_3_ to TbF_3_” QS defines the limits of the “*window* Δ***T***” position on the ***T*** axis in the entire array of *R*F_3_-*R’*F_3_ systems as very wide.

All these studies, especially in recent years, have prepared the formulation of the *chemical design* of *two-component* NTE-II materials with REE trifluorides with *adjustable properties*.

The full range of regulated parameters of fluoride NTE-II materials in the *R*F_3_-*R’*F_3_ systems can be found from the analysis of the phase diagrams of the studied systems and the “from PmF_3_ to TbF_3_” QS [8]. The *R*F_3_-*R’*F_3_ phase diagrams presented in this paper were obtained experimentally. The ratio of the components in them varies from 0 to 100 mol. %.

*The aim of this study* is to predict, using the principle of isovalent isomorphism, *R*F_3_-*R*’F_3_ systems in which two-phase materials with NTE-II are formed.

Research objectives: (1) to identify the stages of the *chemical design* of *R*F_3_-*R’*F_3_ systems for the search for fluoride NTE-II materials; (2) based on the ChCl of *R*F_3_-*R’*F_3_ systems, give a forecast of 50 systems (out of 105) with *two-phase composites* having the *giant* NTE-II at the ***β***-***ss*** → ***t***-***ss*** PolTr (at heating); (3) using the concept of the “from LaF_3_ to LuF_3_” QS [8], determine the range of ***T***_trans_ of components in the area in which the position of the “*window* Δ***T***” on the ***T*** axis in the “from LaF_3_ to LuF_3_” QS is regulated; (4) evaluate the practical applicability of the (***β***-***ss*** +***t***-***ss***) *two-phase composite* NTE-II materials from the kinetics of phase transformations in the subsolidus region of the studied *R*F_3_-*R’*F_3_ systems in which they are formed.

## 2. Results and Discussion

### Four Stages of the Chemical Design of Rare-Earth Fluoride Materials with Adjustable NTE-II Parameters

This approach is called *chemical design* because it uses *high-temperature chemical interactions* in *R*F_3_-*R*’F_3_ systems.

The *chemical design* of materials with NTE-II includes four stages.

The *first stage* involves the choice of “*mother*” substances with the *giant* NTE-II. It is identical to the proposed paradigm [10].

The *density anomaly* at the PolTr is a *necessary* condition for NTE-II. By describing *R*F_3_ as a *single-component* material with NTE-II, we are actually doing this for the first time in the field of materials science.

In the literature, the *coefficients* of a *volumetric* (α_V_) or linear (α_L_) *thermal expansion* CTE are called NTE-II parameters. For the PolTr in *single-component R*F_3_ from the SSGr **B**, the CTE cannot be defined at Δ***T*** = 0. Therefore, the CTE is not a parameter of NTE-II (see below and [10]).

The *second stage* involves the choice of isomorphism as a method of modifying the “*mother*” *R*F_3_. Isomorphism is the most common method for *chemical modification* of properties, including NTE-II.

The *chemical design* by *chemical modification* of simple *R*F_3_ compounds with the *giant* NTE-II by means of *isovalent isomorphism* leads to binary *R*F_3_-*R*’F_3_ systems as the simplest of the multicomponent systems.

*Isovalent isomorphism* is exceptionally effective in modifying the properties of REE trifluorides. Studies on the phase diagrams of 34 *R*F_3_-*R*’F_3_ systems of chemically similar fluorides [1] have shown a wide development of isomorphism and provided digital data for the second stage of the *chemical design*.

The *third stage* of the *chemical design* is new in NTE-II search concepts. It is based on studies of the *high-temperature chemistry* of the *R*F_3_-*R*’F_3_ systems. At this stage, the systems in which NTE-II materials are formed are predicted based on the *chemical interactions of the components* [1].

The possibility of using the *chemical design* is limited for most *homologous series* of REE compounds. The number of *homologous series* of REE compounds with the studied phase diagrams is usually small. It is often combined with an incomplete set of REE. There are no Pm compounds anywhere for the reason described above. The difference in the methods of studying compounds, sometimes separated by decades, reduces the comparability of data from different authors.

The long *R*F_3_ *homological series* of 15 trifluorides (LaF_3_ and 14 *Ln*F_3_ with *Ln* = Ce-Lu) is unique in the completeness of the study of individual trifluorides and the systems formed by them. It is also unique in terms of the high rate of equilibrium in the formation of NTE-II materials (see above).

Fluoride rare-earth NTE-II materials are formed in systems with one or two *R*F_3_ components with the *giant* NTE-II [4,9].

Without ChCl, the analysis of such *R*F_3_-*R*’F_3_ systems is impossible. ChCl, which was recently created [16], is the basis of the *third stage* of the *chemical design* of systems with *multicomponent* NTE-II materials.

The *third stage* is discussed in the following section. For the first time four types of systems (out of 10) in which NTE-II materials are formed were identified in ChCl.

The *fourth stage* of the *chemical design* for the study of materials with NTE-II is used for the first time. Quantitative data forming NTE-II were obtained from the analysis of the position of NTE-II materials on a phase diagram, their phase composition, and equilibrium phase reactions in a binary system. They are the values of the ΔV/V volume change of a two-phase composite in the interval ***T***, determined by the “*window* Δ***T***”.

The “*window* Δ***T***” and the ΔV/V = ***f***(***T***) dependencies are calculated from the phase reaction curves. This stage is based on the thermodynamics of chemical systems, which has not been previously used for the analysis of NTE-II [10].

The proposed scheme for calculating the parameters of NTE-II materials can be applied to each of the studied *R*F_3_-*R*’F_3_ systems with NTE-II. To achieve this, the liquidus and solidus curves of *solid solutions* (***ss***) and the changes in their lattice parameters (V_form_) with composition must be determined in these systems.

The special role of structural changes during the PolTr in the formation of NTE-II materials separates them from *normal* materials possessing NTE-I, in which the growth of ΔV/V occurs with the growth of ***T***.

To date, there is no data on the structural mechanism of NTE-II in *R*F_3_. Its detection should be the *fifth stage* in the *chemical design* of fluoride NTE-II materials.

## 3. Materials and Methods

The *chemical design* of materials with NTE-II among 105 *R*F_3_-*R’*F_3_ systems is based on ChCl [16].

### 3.1. NTE-II and ChCl of the RF_3_-R’F_3_ Systems

**Figure 1:**
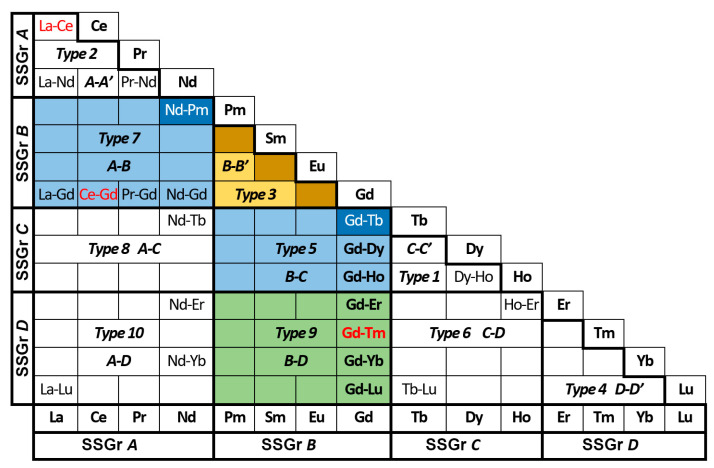
The *chemical design* of 50 (out of 105) systems with the NTE-II materials (shown in color).

ChCl built on the inversely proportional dependency of the degree of ChProx of *R*F_3_ on ΔZ (the difference in Z of cations) [16]. ChCl is represented as a coordinate semi-square, Figure 1.

The coordinate semi-square is a graphical representation of the number of combinations of 15 objects, two in each (C^15^_2_). It is formed by the intersections of 15 columns of the *long homologous series* from LaF_3_ to LuF_3_ (without ScF_3_ and YF_3_) and 14 horizontal rows from CeF_3_ to LuF_3_. The upper right half of the *square* is discarded as a replicate.

There are 105 rectangles left. Each rectangle represents a separate *R*F_3_–*R’*F_3_ system. The studied systems are indicated by the REE symbols.

The *first level* of the ChCl divides 15 *R*F_3_ by *structural features* (a type of structure and the presence of the PolTr) into four *structural subgroups* (SSGrs *A–D*) (Table 1). We highlighted the SSGr ***B*** in bold, emphasizing the presence of the PolTr with NTE-II.

The *second level* of the ChCl comprises 10 TypeSs. The types are paired combinations of four SSGrs: 1 *C-C’*, 2 *A-A*’, 3 ***B-B’***, 4 *D-D’*, 5 ***B****-C*, 6 *C-D*, 7 *A-**B***, 8 *A-C*, 9 ***B****-D*, 10 *A-D* (arranged in ascending order of |ΔZ_max_| in Figure 1).

In the *third level*, four *groups of systems* (**GrSs**) are formed from TypeSs with a certain |ΔZ_max_| [1,16].

The GrSs and TypeSs include several systems. To compare the TypeSs and GrSs, two values of the ChProx in each are used: maximum |ΔZ_max_| and minimum |ΔZ_min_| (Table 2) [1,16]. Only |ΔZ_max_| is discussed here.

The third level of the GrSs is partially shown in Figure 1 in color: for GrS-1 in *yellow* and *dark yellow*, for GrS-2 in *blue* and *dark blue*, and for GrS-3 in green (the colors are the same as in the ChCl in [16]).

Six TypeSs remained colorless in Figure 1. These TypeSs are divided into two groups. Four TypeSs belong to the first group: TypeS-1 (*C-C’*), TypeS-2 (*A-A’*), TypeS-4 (*D-D’*) and TypeS-6 (*C-D*). The Z_av_ of *R* from the SSG ***B*** cannot be realized in these SSGs. These systems do not contain materials with NTE-II.

The *third stage* of the *chemical design* allocates 50 *R*F_3_-*R’*F_3_ systems (Figure 1) with *R*F_3_ from the SSGr ***B*** (*R* = Pm, Sm, Eu, and Gd) as the sources of fluoride materials with adjustable NTE-II parameters. Only four TypeSs (TypeS-3, TypeS-5, TypeS-7, TypeS-9) (out of 10) contain *R*F_3_ with the ***β***- → ***t***- PTs (at heating) with NTE-II.

Table 2 lists the number of systems with NTE-II materials in each TypeS.

### 3.2. NTE-II Materials in TypeS-3 (**B**-**B**’) from RF_3_ of the SSGr **B**

The TypeS-3 (***B-B’***) from the components of the SSGr ***B*** includes six systems, as shown in Table 2 and Figure 1.

According to the ChProx these components are heterogeneous. Three systems, PmF_3_-SmF_3_, SmF_3_-EuF_3_, and EuF_3_-GdF_3_, were distinguished from the others in the TypeS-3 by the maximum ChProx |ΔZ| = 1 of the components. They are marked in *dark yellow* in Figure 1.

The TypeS-3 (***B***-***B’***) includes six systems. None have been studied. Of these, three systems (PmF_3_-SmF_3_, SmF_3_-EuF_3_, and EuF_3_-GdF_3_) are special “synthetic” systems. It is composed of *R*F_3_-*R’*F_3_ systems with ΔZ = 1.

The *full* QS was described in [8]. It contains 14 systems of 105 *R*F_3_-*R*’F_3_. Let us call these systems *particular*. The *full* QS is composed of LaF_3_-CeF_3_, CeF_3_-PrF_3_, PrF_3_-NdF_3_, NdF_3_-PmF_3_, PmF_3_-SmF_3_, SmF_3_-EuF_3_, EuF_3_-GdF_3_, GdF_3_-TbF_3_, TbF_3_-DyF_3_, DyF_3_-HoF_3_, HoF_3_-ErF_3_, ErF_3_-TmF_3_, TmF_3_-YbF_3_, and YbF_3_-LuF_3_ systems. “*Particular*” indicates that the condition |ΔZ| = 1 is “bound” to a system in this context. Only strictly defined systems that obey this condition can be adjacent to QS.

From *full* QS stands out the *section* of SSGr ***B***. Let us call it *short* QS “from PmF_3_ to GdF_3_(TbF_3_)”. These systems are marked in *dark yellow* in Figure 1. This *short* QS is used to analyze the dependence of the position of *R*F_3_ (*R* = Pm-Tb) ***T***_trans_.

**Figure 2:**
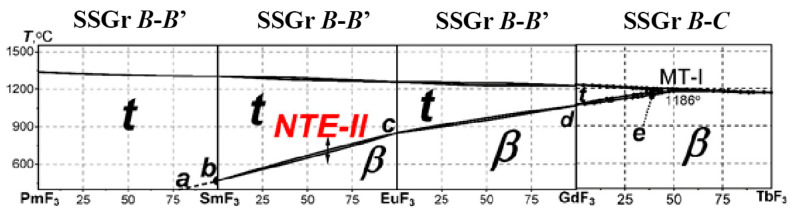
The *chemical design* of ***T***_trans_ in the *short* QS “from PmF_3_ to GdF_3_(TbF_3_)”. Curve ***a-b-c-d-e***, in the *short* “from PmF_3_ (point ***a***) to GdF_3_ (Gd_0.57_Tb_0.43_F_3_, point ***e***)” QS presents the dependence of ***T***_trans_ on Z.

Three *particular systems* of the *short* QS are shown in Figure 2. NTE-II during the PolTr in the SmF_3_-EuF_3_ system is indicated by an arrow. Similar PolTrs exist in two adjacent systems PmF_3_-SmF_3_ and EuF_3_-GdF_3_.

The PmF_3_-EuF_3_, SmF_3_-GdF_3_, and PmF_3_-GdF_3_ systems of the TypeS-3 have |ΔZ| > 1. They do not belong to the *short* QS, but are included in the list of systems from the TypeS-3 with NTE-II materials (Table 3). *Continuous* ***ss*** are formed between their components. The structural modifications of the components involved in the PolTrs cause NTE-II. These systems are marked in *yellow* in Figure 1.

In Figure 2, the MorphTr in the GdF_3_-TbF_3_ system is added to the two PolTrs considered. This is the *true* MorphTr (according to V.M. Goldschmidt [11]). This is the MorphTr of the first type (**MorphTr**-1) between the ***β***-***ss*** and ***t***-***ss***. The appearance of the MorphTr-1 in the GdF_3_-TbF_3_ system is caused, as in *R*F_3_, by a change in the structure type. The change proceeds in accordance with the mechanism of the MorphTr, an *invariant* (***T*** = *const*) *peritectic phase reaction* with the melt (***Liq***) [1]:

***β***-Gd_0.49_Tb_0.51_F_3_ ↔ ***Liq*** (melt) + ***t-***Gd_0.57_Tb_0.43_F_3_

### 3.3. NTE-II Temperature Control in the Short “from PmF_3_ to TbF_3_” QS

The position of the ΔV/V_form_ jump of Δ***T*** on the ***T*** axis plays a critical role for the practical use of NTE-II materials. Most applications require room temperature. In the *short* “from PmF_3_ to TbF_3_” QS Δ***T***_trans_ can be controlled by the *R*F_3_-*R’*F_3_ systems.

Dimorphic *R*F_3_ (*R* = Pm-Gd) have a uniquely large range of variation in ***T***_trans_ during the PolTrs. In *R*F_3_-*R’*F_3_ systems, there is a possibility of more “subtle” control of Δ***T***_trans_ by *isomorphic substitutions*.

The limit of ***T***_trans_ values for *two-component* materials with NTE-II is determined by the *short* QS of the *full* QS which contains *R*F_3_ of the SSGr ***B*** (*R* = Pm, Sm, Eu, and Gd). Curve ***a-b-c-d-e***, Figure 2, in the *short* “from PmF_3_ (point ***a***) to GdF_3_ (Gd_0.57_Tb_0.43_F_3_, point ***e***)” QS presents the dependence of ***T***_trans_ on Z. Materials with this Δ***T***_trans_ and Z have NTE-II.

With the help of the *chemical design* of NTE-II materials in *R*F_3_-*R’*F_3_ systems, ***T***_trans_ can be changed from −148 °C in PmF_3_ [9] to 1186 ± 10 °C for the MorphTr-1 with ***t-***Gd_0.57_Tb_0.43_F_3_ [1].

According to [10], the “*window* Δ***T***” of known NTE-II materials does not exceed 600 °C. Fluoride NTE-II materials surpass it by almost three times.

Fluoride NTE-II materials have a problem of accessibility when they need to be used at standard (room) ***T***. The continuation of the curve ***a***-***b*** in Figure 2 to the room ***T*** region corresponds to the PmF_3_-SmF_3_ system with an inaccessible PmF_3_.

This problem is solved by the wide possibilities of carrying out *structural* and *chemical* modeling of the “lanthanide” for any average value of the *atomic number* Z_av_ between ^58^Ce and ^71^Lu [9]. The necessary *short* QS is selected for modeling. The model composition with Z_av_ can be calculated for the required ***T***_trans_.

*Structural* and *chemical modeling* of “lanthanides” with an intermediate Z_av_ moves the NTE-II “*window* Δ***T***” of the adjustable interval on the ***T*** scale (see above). This provides a solution to the availability problem (PmF_3_) and high cost of some REE, allowing the replacement of unavailable *R*F_3_.

The temperature range in which the *chemical design* can adjust ***T***_trans_ in *R*F_3_ follows from the *short* “from PmF_3_ to GdF_3_ (Gd_0.57_Tb_0.43_F_3_)” QS (the curve ***a***–***e***, Figure 2). The isomorphism continuously changes the ***T***_trans_ of the *R*_1-x_*R’*_x_F_3_ PolTrs from (−148 °C) in PmF_3_ (and lower in the unexplored part of the NdF_3_-PmF_3_ system) up to 1186 ± 10 °C for MorphTr-1 with ***t***-Gd_0.57_Tb_0.43_F_3_.

In the other three systems, |ΔZ_max_| increases to 2 and 3. This does not limit the formation of phases with NTE-II in the systems of the TypeS-3.

### 3.4. NTE-II Materials in the TypeS-5 (**B**-C), TypeS-7 (A-**B**), and TypeS-9 (**B**-D)

Let us arrange the TypeSs with the SSGs components in increasing order |ΔZ_max_| (Table 2): the TypeS-5 (***B****-C*) |ΔZ_max_| = 6, TypeS-7 (*A-**B***) |ΔZ_max_| = 7, and TypeS-9 (***B****-D*) |ΔZ_max_| = 10.

#### 3.4.1. *NTE-II Materials in the TypeS-5 (**B**-C)*

**Figure 3:**
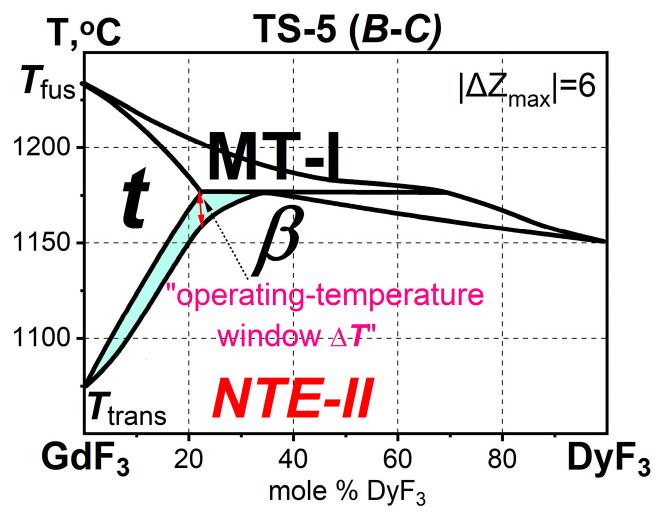
The GdF_3_-DyF_3_ system of the TypeS-5 (***B****-C*) with the ***β****-****ss*** →***t-ss***PolTr with NTE-II. Δ***T*** > 0 is marked with a red arrow. The two-phase (***t-*** + ***β****-*) region is highlighted in blue.

There are 12 systems in the TypeS-5 (Table 4). The phase diagrams of the GdF_3_-TbF_3_ and GdF_3_-DyF_3_ systems are shown in Figure 2 and Figure 3. For the TypeS-5 |ΔZ_max_| = 6, which is twice that for the TypeS-3.

This decrease in the ChProx of *R*F_3_ causes, starting with the GdF_3_-TbF_3_ system, the emergence of a new topological feature, MorphTr-1 (the *peritectic phase reaction*). The appearance of MorphTr-1 (Table 2) follows the critical |ΔZ_max_| [1].

The GdF_3_-DyF_3_ system (Figure 3) from the TypeS-5 (***B****-C*) contains the *limited* ***t-***Gd_1-x_Dy_x_F_3_ and *continuous* ***β-***Gd_1-x_Dy_x_F_3_ ***ss*** [1]. The “*window* Δ***T*** > 0” is marked with a red arrow.

#### 3.4.2. *NTE-II Materials in the TypeS-7 (A-**B**)*

The TypeS-7 contains 16 systems (Table 5) with |ΔZ_max_| = 7 from *R*F_3_ of different SSGrs.

In the NdF_3_-PmF_3_ system, the first component is the last in the SSGr *A*, and the second component is the first component in the subsequent SSGr ***B***. As a result, the NdF_3_-PmF_3_ system has |ΔZ| = 1, which refers to the QS. This system is borderline, similar to the GdF_3_-TbF_3_ system. The phase diagram of this system has not yet been studied.

**Figure 4:**
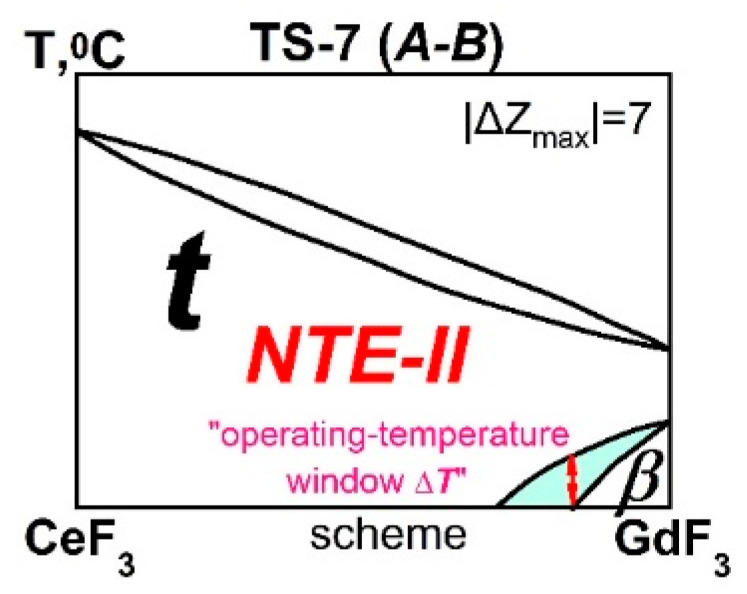
The scheme of the CeF_3_-GdF_3_ system of the TypeS-7 (*A*-***B***) with NTE-II materials. The two-phase (***t-*** + ***β****-*) region is highlighted in blue.

The high |ΔZ_max_| makes ***β-ss*** limited, leaving *continuous* ***t-ss*** at high temperatures (Figure 4 and Table 2).

#### 3.4.3. *NTE-II Materials in the TypeS-9 (**B**-D)*

The TypeS-9 consists of 16 systems with one component from the SSGr ***B*** (Table 6). In this TypeS, |ΔZ_max_| reaches 10. A large chemical difference in the Z values of the components is manifested in the difference in the structural types of the components. In the SSGr *D*, to which the second components of the TypeS-9 systems belong, the ***α***-type appears at high temperatures.

MorphTr-2 of the second type, ***Liq*** (melt) →***β-*** + ***α-*** (*eutectic phase reaction*), is also accompanied by a *giant* ΔV/V_form_ jump. In MorphTr-2, there is an increase in volume of the high-temperature ***α-***modification by 16% (to the smaller value).

At |ΔZ_max_| = 10 in TypeS-9, the chemical interactions of the components lead to the simultaneous appearance of two topological signs of strong interactions: MorphTrs of two types.

**Figure 5:**
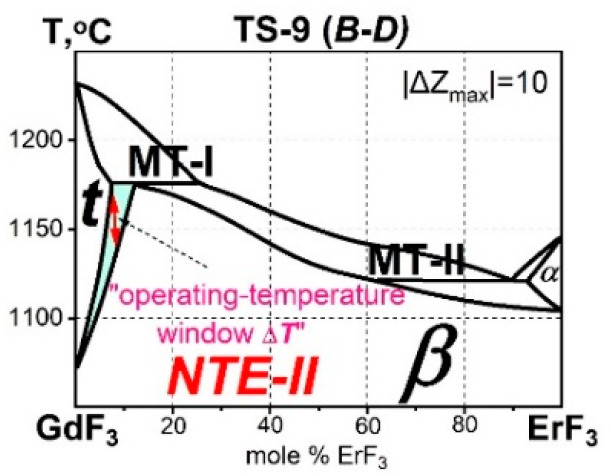
The GdF_3_-ErF_3_ system from the TypeS-9 (***B****-D*) with NTE-II materials. The two-phase (***t-*** + ***β****-*) region is highlighted in blue.

In the phase diagram of the GdF_3_-ErF_3_ system (Figure 5), only one structural transformation, MorphTr-1 ***β-ss*** → ***t-ss*** (at heating), is associated with NTE-II. The peritectic MorphTr-1 that originated in the TypeS-5 (***B****-C*) is supplemented by the eutectic MorphTr-2 in the GdF_3_-ErF_3_ system from the TypeS-9 (***B****-D*). This MorphTr-2 is not related to NTE-II.

The *chemical design* with the ChCl highlights 50 systems (out of 105) of the four TypeSs in which the presence of NTE-II materials is possible. One or both components of these systems are selected from dimorphic *R*F_3_ from the SSG ***B***(*R* = Pm, Sm, Eu, and Gd). They have the ***β-ss*** → ***t-ss*** PolTrs (at heating) associated with NTE-II. Only 11 of the 50 systems were studied. They are shown in **bold** in Table 3, Table 4, Table 5 and Table 6.

### 3.5. NTE-II Materials in the TypeS-8 (A-C) and TypeS-10 (A-D)

The remaining TypeS-8 (*A-C*) and TypeS-10 (*A-D*) implement Z_av_, corresponding to the Zs of *R*, which form fluorides from the SSGr***B*** (from Pm to Gd). The first TypeS-8 has |ΔZ_max_| = 10 (similar to TypeS-9). The second has the highest |ΔZ_max_| = 14 for all TypeS. The phase diagrams at high |ΔZ_max_| become very complicated.

### 3.6. NTE-II and Kinetics of Phase Formation in RF_3_-R’F_3_ Systems

The features of NTE-II materials relate only to the equilibrium state. Structural transformations of the phases with NTE-II along the curves of their decay (formation) occur in the solid state. To achieve equilibrium, disinhibited changes in the phase composition are required.

The processes of ***ss*** ordering in the subsolidus and the formation of double compounds are usually inhibited. There are no such processes in the studied *R*F_3_-*R*’F_3_ systems [1].

“Liquid–solid” transitions are disinhibited. In the 34 studied systems, the thermal effects of crystallization (liquidus and solidus) were well separated by thermal analysis [1]. According to the kinetics of melt crystallization of the studied *R*F_3_-*R’*F_3_ systems during thermal analysis and the thermal effects of phase transformations, *two-phase composite* NTE-II materials, ***β***-***ss*** +***t***-***ss***, formed during phase reactions are in equilibrium with a high probability.

In this paper, 50 (out of 105) *R*F_3_-*R*’F_3_ systems in which these materials may form were selected using the principle of isovalent isomorphism.

Composite materials with NTE-II are also formed in *M*F_2_-*R*F_3_ systems with *M* = Ca, Sr, Ba; *R* = Gd-Lu, Y, and NaF-*R*F_3_ with *R* = Gd, Tb [1]. The LaF_3_-type structure has a high isomorphic capacity (up to 35% mol.) in relation to REE ions. When part of the *R*^3+^ cations is replaced with *M*^2+^ (Na^+^) cations (aliovalent isomorphism), berthollide phases with the ***t***-type structure are formed [1,17,18]. When the temperature decreases, their decay occurs over the temperature range (the ***t***- → ***β***- PolTr with “window ΔT” > 0) with the formation of ***β***-type phases [1]. The thermodynamic aspects of the phase transitions in the NTE-II berthollide phases are not included in the objectives of this study.

The ***t***-type *R*F_3_ and *R*_1-x_*R*’_x_F_3_ considered in this report and berthollide phases based on *R*F_3_ can be obtained in the form of single crystals [1]. They are the best superionic conductors with fluorine-ion conductivity [19,20,21,22,23,24,25]. They are used as lasers [26,27] and as scintillators [28,29,30,31,32,33]. Nonstoichiometric crystals with the ***t***-type structure have a low refractive index dispersion at the level of the initial *R*F_3_ [34].

## Figures and Tables

**Table 1:** The SSGrs *A–D* of *R*F_3_ in the ChCl [1,16].

SSGrs	*R*F_3_	Structural Types of *R*F_3_	Abbreviations
*A*	LaF_3_, CeF_3_, PrF_3_, NdF_3_	The high-temperature LaF_3_ ***t***-type up to the melt	***t-***
***B***	***Dimorphic trifluorides*:** **PmF_3_, SmF_3_, EuF_3_, GdF_3_**	(1) ***t****-*type up to the melt, (2) The low temperature β-YF_3_ ***β***-type	***t-*** ***β-***
*C*	TbF_3_, DyF_3_, HoF_3_	***β-***type up to the melt	***β-***
*D*	*Dimorphic trifluorides*: ErF_3_, TmF_3_, YbF_3_, LuF_3_	(1) α-YF_3_ ***α***-type up to the melt, (2) ***β-***type	***α-*** ***β-***

*R*F_3_ from the the SSGr ***B*** are highlighted in **bold**.

**Table 2:** Four TypeSs with components from the SSGr ***B*** with NTE-II materials.

TypeS-N (*B*-*X*) *X* = *A, B, C, D*	|ΔZ|_max_/|ΔZ|_min_	SSGr	Topological Signs of Phase Diagrams	Systems with NTE-II
TypeS-3 (***B-B’***)	3/1	***B*** = **PmF_3_**, **SmF_3_**, **EuF_3_**, **GdF_3_**	Unlimited ***t-ss*** and ***β-ss***	6
TypeS-5 (***B****-C*)	6/1	*C* = TbF_3_, DyF_3_, HoF_3_	Unlimited ***β-ss*** and limited ***t-ss*** + MorphTr-1 (peritectic)	12
TypeS-7 (*A-**B***)	7/1	*A* = LaF_3_, CeF_3_, PrF_3_, NdF_3_	Unlimited ***t-ss*** + limited ***β-ss***	16
TypeS-9 (*B-D*)	10/4	*D* = ErF_3_, TmF_3_, YbF_3_, LuF_3_	Limited ***t*-*ss*** + unlimited ***β*-*ss*** (MorphTr-1 + MorphTr-2)	16

*R*F_3_ from the the SSGr ***B*** are highlighted in **bold**.

**Table 3:** The TypeS-3 (***B-B’***) with the ***β-ss*** → ***t***-***ss*** PolTs and NTE-II.

No.	*B*-*B*’	|ΔZ|
1	PmF_3_-SmF_3_	1
2	SmF_3_-EuF_3_	1
3	EuF_3_-GdF_3_	1
4	PmF_3_-EuF_3_	2
5	SmF_3_-GdF_3_	2
6	PmF_3_-GdF_3_	3

**Table 4:** 12 systems of the TypeS-5 (***B****-C*) with the ***β****-*ss → t***-ss*** PolTrs and NTE-II.

No.	*B*-*C*	|ΔZ|	No.	*B*-*C*	|ΔZ|
1 *	**GdF_3_-TbF_3_**	1	7	PmF_3_-TbF_3_	4
2	EuF_3_-TbF_3_	2	8	SmF_3_-DyF_3_	4
**3**	**GdF_3_-DyF_3_**	2	9	EuF_3_-HoF_3_	4
4	SmF_3_-TbF_3_	3	10	PmF_3_-DyF_3_	5
5	EuF_3_-DyF_3_	3	11	SmF_3_-HoF_3_	5
**6**	**GdF_3_-HoF_3_**	3	12	PmF_3_-HoF_3_	6

* The studied systems are shown in **bold**.

**Table 5:** 16 systems of the TypeS-7 (*A-**B***) with the ***β****-**ss*** → ***t-ss*** PolTrs and NTE-II.

No.	*A*-*B*	|ΔZ|	No.	*A*-*B*	|ΔZ|
1	NdF_3_-PmF_3_	1	9	PrF_3_-EuF_3_	4
2	PrF_3_-PmF_3_	2	10 *	**NdF_3_-GdF_3_**	**4**
3	NdF_3_-SmF_3_	2	11	LaF_3_-SmF_3_	5
4	CeF_3_-PmF_3_	3	12	CeF_3_-EuF_3_	5
5	PrF_3_-SmF_3_	3	**13**	**PrF_3_-GdF_3_**	**5**
6	NdF_3_-EuF_3_	3	14	LaF_3_-EuF_3_	6
7	LaF_3_-PmF_3_	4	**15**	**CeF_3_-GdF_3_**	**6**
8	CeF_3_-SmF_3_	4	**16**	**LaF_3_-GdF_3_**	**7**

* The studied systems are shown in **bold**.

**Table 6:** The TypeS-9 (***B****-D*) from 16 systems with the ***β-ss*** → ***t-ss*** PolTr and NTE-II.

No.	*B*-*D*	|ΔZ|	No.	*B*-*D*	|ΔZ|
1 *	**GdF_3_-ErF_3_**	**4**	9	EuF_3_-YbF_3_	7
2	EuF_3_-ErF_3_	5	**10**	**GdF_3_-LuF_3_**	**7**
**3**	**GdF_3_-TmF_3_**	**5**	11	PmF_3_-TmF_3_	8
4	SmF_3_-ErF_3_	6	12	SmF_3_-YbF_3_	8
5	EuF_3_-TmF_3_	6	13	EuF_3_-LuF_3_	8
**6**	**GdF_3_-YbF_3_**	**6**	14	PmF_3_-YbF_3_	9
7	PmF_3_-ErF_3_	7	15	SmF_3_-LuF_3_	9
8	SmF_3_-TmF_3_	7	16	PmF_3_-LuF_3_	10

* The studied systems are shown in **bold**.

## Data Availability

The data presented in this study are available on request from the corresponding author.

## Abbreviations

PolTr	Polymorphic transformation
NTE	Negative thermal expansion
NTE-II	Negative thermal expansion based on a phase-transition-type mechanism
QS	Quasi-system
***ss***	Solid solution
ChProx	Chemical proximity
ChCl	Chemical classification
TypeS	Type of a system
GrSs	Groups of systems
REE	Rare-earth element
V_form_	Volume of one formula unit
SSGr	Structural subgroup
MorphTr	Morphotropic transformation
